# Regulatory Mechanisms of Baicalin in Cardiovascular Diseases: A Review

**DOI:** 10.3389/fphar.2020.583200

**Published:** 2020-11-02

**Authors:** Laiyun Xin, Jialiang Gao, Hongchen Lin, Yi Qu, Chang Shang, Yuling Wang, Yingdong Lu, Xiangning Cui

**Affiliations:** ^1^ The First Clinical Medical College, Shandong University of Traditional Chinese Medicine, Jinan, China; ^2^ Department of Cardiology, Guang’ anmen Hospital, China Academy of Chinese Medical Sciences, Beijing, China

**Keywords:** baicalin, cardiovascular diseases, inflammation, oxygen reactive species, apoptosis, immunomodulatory

## Abstract

Cardiovascular diseases (CVDs) is the leading cause of high morbidity and mortality worldwide, which emphasizes the urgent necessity to develop new pharmacotherapies. In eastern countries, traditional Chinese medicine *Scutellaria baicalensis* *Georgi* has been used clinically for thousands of years. Baicalin is one of the main active ingredients extracted from Chinese herbal medicine *S. baicalensis*. Emerging evidence has established that baicalin improves chronic inflammation, immune imbalance, disturbances in lipid metabolism, apoptosis and oxidative stress. Thereby it offers beneficial roles against the initiation and progression of CVDs such as atherosclerosis, hypertension, myocardial infarction and reperfusion, and heart failure. In this review, we summarize the pharmacological features and relevant mechanisms by which baicalin regulates CVDs in the hope to reveal its application for CVDs prevention and/or therapy.

## Introduction

Cardiovascular diseases (CVDs) have become the leading cause of disability and death on a global scale (Timmis et al., 2018; Virani et al., 2020). CVDs lead to nearly one in three deaths in developed countries and one of every four deaths in developing countries according to epidemiological studies (Heidenreich et al., 2011; Zhao D. et al., 2019). Given the unmet needs in CVD control from Western medicine (Aggarwal et al., 2017), a complementary and alternative approach for treatment of CVD is needed. Traditional Chinese medicine (TCM), owning a history of 2,000 years, has drawn growing attention from the cardiovascular research community due to its their “multiple targets and multiple channels”. Importantly, according to current guidelines, Chinese patients with coronary heart disease tend to use less Western medicine while applying more types of TCM (Guo et al., 2013). Moreover, TCM is increasingly welcomed in many developed countries, including the United States and Australia.

**Figure 1:**
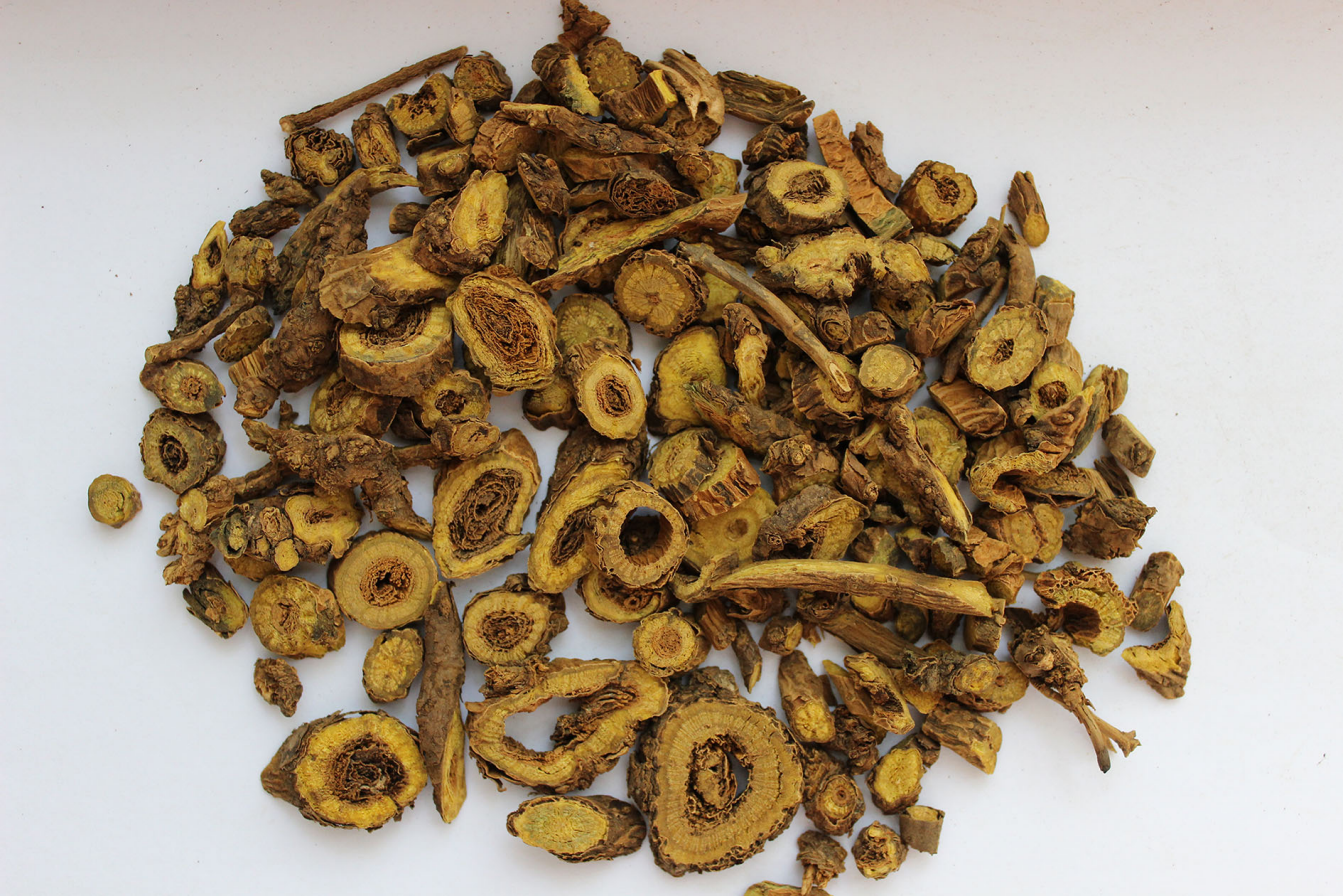
The medicinal part of *Scutellaria baicalensis* Georgi and the main extraction source of baicalin.

Baicalin is a monomeric flavonoid compound extracted from the root of *Scutellaria baicalensis Georgi* (SBG), a species of flowering plant in the Lamiaceae family (Chen et al., 1994). Baicalin is found in the root (10.11%), which is the main medicinal part of SBG (**Figure 1**) (Shang et al., 2010; Dinda et al., 2017). SBG is an important ingredient in Xiaochaihu preparations, Gegenqinlian decocation and Sho-saiko-to preparations whose extracts include a key component of baicalin (Zhang, 1974; Srinivas, 2010; Zhao et al., 2016). Previous studies have high-lighted the extensive pharmacological properties of baicalin for treating CVDs such as atherosclerosis (AS), hypertension, and ischemic heart disease. Considering its therapeutic mechanism may be associated with hypolipidemic, anti-inflammatory, anti-oxidant, anti-apoptosis properties (Chen et al., 2015; He et al., 2016; Dai et al., 2017) and considering its pharmacological properties and therapeutic potentials in the progression of CVDs, herein, we summarize and generalize the regulatory mechanisms involving baicalin in CVDs pathogenesis with a view to provide a reference for the development of new drugs for the treatment of CVDs.

## Pharmacological Features of Baicalin

### Bioactive Components in SBG

**Figure 2:**
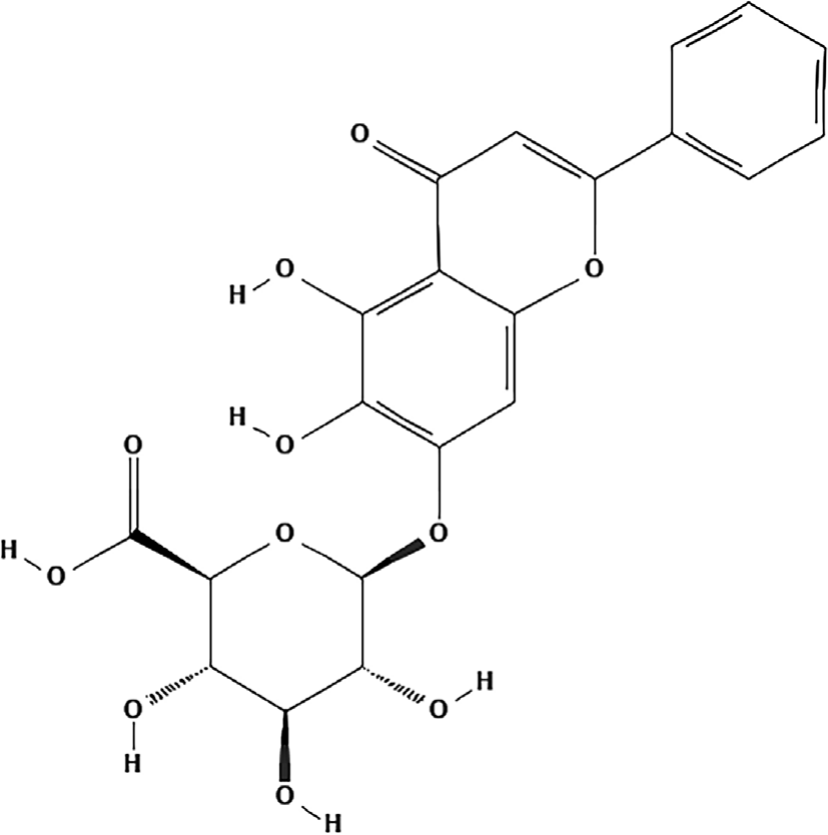
The chemical structure of baicalin.

Despite the popular clinical use of SBG, its active ingredients and the molecular mechanisms have not been fully clarified in detail to date. It has been reported that extracts of SBG and its major chemical constituents possess anti-viral, anti-tumor, antioxidant, anti-inflammatory, and neuroprotective activities (Wang Z. L. et al., 2018). With the deepening of understanding, increasing attention paid by the cardiovascular research community is focused on the bioactive chemical monomers comprising SBG that are responsible for its pharmacological activities. To date, many chemical constituents such as flavonoids, volatile oils, terpenoids, polysaccharides, steroids and amides in SBG have been isolated and identified. Flavonoids and their glycosides are considered to be characteristic components of SBG. According to the literature research, the most representative ingredients are baicalin, baicalein, wogonoside and wogonin (Li C. et al., 2011; Zhao T. et al., 2019). Baicalin has poor solubility in water and lipid. In terms of the involvement of these chemicals in the development of agents for the treatment of CVDs, baicalin (C_21_H_18_O_11_) is not only the first reported and the most abundant component but it also the component with greatest potential and value of those investigated (**Figure 2**) (Azimova and Vinogradova, 2013; Zhao T. et al., 2019).

### Pharmacokinetics of Baicalin

Based on some advanced detection methods, pharmacokinetic investigations involving baicalin have been broadly carried out in the past few years. Absorption studies indicate that baicalin is poorly absorbed from the gastrointestinal tract in its native form and must be transformed into its aglycon baicalein through the hydrolysis by the intestinal bacteria, then baicalein can be restored to baicalin by UDP-glucuronosyltransferase (UGT) in the systemic circulation (Lu et al., 2007; Noh et al., 2016; Zhang et al., 2017). Since baicalein is optimally absorbed in all segments of the gastrointestinal tract, the key step in the absorption process of baicalin is the conversion of baicalin to baicalein (Huang et al., 2019). Furthermore, baicalin showed bimodal or multiple peaks in its absorption profile (Huang et al., 2012; Zhao et al., 2014; Zhang J. et al., 2016). The first peak was reported to occur within 45 min of ingestion, which was probably due to direct absorption, while the second peak occurred after 8–12 h [intravenously (i.v.)] or 12–24 h [orally (p.o.)] and was probably associated with the enterohepatic circulation (Lu et al., 2007; Huang et al., 2019). Next in terms its distribution, the binding rate of baicalin and plasma protein was found to range from 86–92%, and the high plasma protein-binding rate allows baicalin to be absorbed rapidly into the plasma (Tang et al., 2006). A tissue distribution study showed that baicalin was found to accumulate in various tissues and was highest in the kidneys after injection (Wei et al., 2016). Lastly, the metabolism and excretion of baicalin follows extensive metabolization by the liver and kidneys and is excreted in bile in the form of glucuronides. After oral administration of baicalin, the total cumulative amount of its glucuronides was determined to be about 54% of the dose (Abe et al., 1990). The major active metabolic sites are the hydroxyl groups on the A ring and 8- and 4’-positions of baicalin and several metabolic enzymes like β-glucuronidase, UGT, sulfatase and catechol-O-methyltransferases are also involved (Wang et al., 2012; Lu et al., 2012; Akao et al., 2013). Data have shown that the elimination half-life of baicalin is 0.1 till 4 h post dose (i.v) and 12.1 h (oral administration) (Xing et al., 2005). Therefore, its insolubility in water and lipid, extensive metabolism and high biliary excretion may contribute to the low bioavailability and short half-life of baicalin. Moreover, pathological conditions may alter the functions of many enzymes and transporters *in vivo*, thus baicalin may exhibit different pharmacokinetic properties under different pathological conditions (Xing et al., 2005; Huang et al., 2019). For instance, baicalin plasma concentration was higher in type 2 diabetic rats than normal rats (Liu et al., 2010). In cerebral ischemia-reperfusion rats, absorption of baicalin was enhanced, whereas it decreased in febrile rats (Ma et al., 2012; Huo et al., 2017).

### Drug Delivery Systems

As a result of the low bioavailability of baicalin, this seriously affected its clinical development, although many new delivery strategies have been designed and developed, including nanonization technology, phospholipid complexes, solid dispersion, inclusion complexes, and micelles (Li B. et al., 2011; Li N. et al., 2011; Yue et al., 2013; Zhang H. et al., 2016; Li et al., 2017). When compared to baicalin alone, modified preparations can improve its dissolution and solubility, which in turn further improve its targeting ability and therapeutic efficacy. For example, the baicalin liposomes modified with folic acid and polyethylene glycol (PEG) can improve the cellular uptake rate and tumor targeting, as well as extend the retention time in the body (Chen et al., 2016). Baicalin liposomes are also known to strengthen oral bioavailability and tissue distribution (Wei et al., 2014). Nonetheless, to date, limited information is available relative to baicalin drug delivery systems for AS therapy. Given the potential clinical applicability of baicalin in the future, suitable vehicles loading baicalin for use in alleviating AS progression deserve further evaluation.

## Molecular Mechanisms and Therapeutic Potential of Baicalin in AS, Myocardial Ischemia–Reperfusion Injury, Hypertension, and Heart Failure

### Atherosclerosis

**Figure 3:**
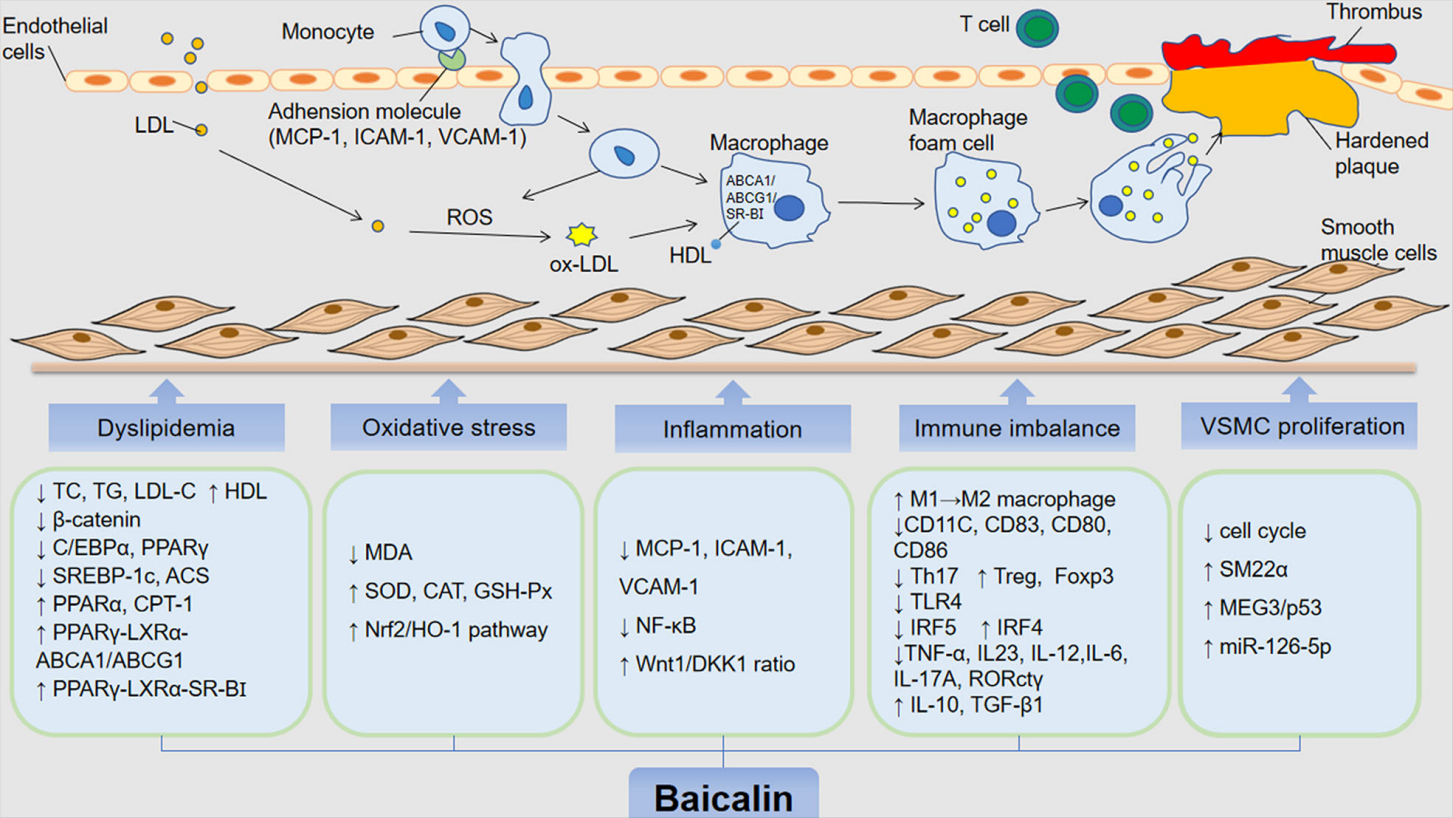
The anti-atherosclerotic effects by which baicalin alleviates the development of AS. The role of baicalin in inhibiting atherosclerosis includes hypolipidemic effects and inhibition of foam cell formation, inhibition of oxidative stress, mitigation of inflammatory response, immunomodulatory, suppression of VSMC proliferation and migration. low density lipoprotein (LDL); oxidized LDL (oxLDL); oxygen reactive species (ROS); high-density lipoprotein (HDL); total cholestero (TC); triacylglycerol (TG); low-density lipoprotein cholesterol (LDL-C); CCAAT/enhancer bindingproteins α (C/EBP α); peroxisome proliferator-activated receptor γ (PPAR γ); liver receptor X (LXR α); ATP binding cassette (ABC) transporter A1/G1 (ABCA1/G1); scavenger receptor BI (SR-BI); sterol regulatory element-binding protein 1c (SREBP-1c); Acyl-CoA synthase (ACS); peroxisome proliferator-activated receptor α (PPAR α); carnitine palmitoyltransferase 1 (CPT-1); malondialdehyde (MDA); superoxide dismutase (SOD); catalase (CAT); glutathione peroxidase (GSH-Px); nuclear factor E2-associated factor 2 (Nrf2); heme oxygenase-1 (HO-1); monocyte chemoattractant protein 1 (MCP-1); intercellular adhesion molecule 1 (ICAM-1); vascular adhesion molecule 1 (VCAM-1); nuclear factor kappa B (NF-κB); dickkopf-related protein-1 (DKK 1); interleukin 10/12/23/6/17A (IL-10); interferon regulatory factor 4 and 5 (IRF4/5); tumor necrosis factor (TNF)-α; transforming growth factor-β1 (TGF-β1); toll-like receptor (TLR4); smooth muscle (SM) 22α; factor forkhead box protein P3 (Foxp3); maternally expressed gene 3/p53 (MEG3/p53).

AS is the primary pathological basis of CVDs, which could lead to dramatic clinical events, such as unstable angina or myocardial infarction (MI) (Reiner et al., 2011). The underlying pathophysiological mechanisms of AS involve endothelial dysfunction, lipid deposition, oxidative stress damage, immune inflammatory response, and platelet migration and aggregation (Packard et al., 2009; Weber and Noels, 2011; Gargiulo et al., 2016; Negre-Salvayre et al., 2017; Wolf and Ley, 2019). Accumulating studies indicate that baicalin can exert protective effects against AS by targeting these proatherogenic processes (**Figure 3**).

#### Hypolipidemic Effects and Inhibition of Foam Cell Formation

AS is a lipid-driven inflammatory disease of the arterial intima (Bäck et al., 2019). Lipid retention in the endothelium exacerbates the permeability of the vascular wall to promote the oxidation and deposition of lipoproteins which trigger the atherosclerotic cascade (Olofsson and Borèn, 2005). Highly effective lipid-lowing drugs are widely used in AS. A clinical trial has shown that baicalin could improve the state of blood lipid disorders and showed better improvements in patients with coronary artery disease, which was associated with decreasing the levels of serum total cholesterol (TC), triacylglycerol (TG), low-density lipoprotein cholesterol (LDL-C) levels and apolipoproteins. Furthermore, in ApoE models, baicalin decreased the lipid profile of TC, TG and LDL-C and ameliorated the progression of AS in a dose-dependent manner (Lee et al., 2009; Hang et al., 2018). Several biological processes associated with baicalin are involved in lipid regulation activities. In terms of the molecular mechanisms involved in baicalin-modulated lipid regulation activities, *an vitro* study of 3T3-L1 preadipocytes determined that baicalin downregulated the expression levels of major transcription factors of adipogenesis including CCAAT/enhancer binding proteins (C/EBPα) and peroxisome proliferator-activated receptor (PPAR) γ (Lee et al., 2009). β-Catenin, upstream of PPARγ and the C/EBPα regulator, has a negative relationship with adipogenesis. Baicalin significantly attenuated β-catenin expression in an *in vitro* study (Lee et al., 2010). Furthermore, another study indicated that baicalin could lower TG levels by favoring lipid oxidation by blocking the expression of sterol regulatory element-binding protein 1c (SREBP-1c) and Acyl-CoA synthase (ACS) apart from increasing the expression of lipolysis-related proteins such including PPARα and carnitine palmitoyltransferase 1 (CPT-1) (Wu et al., 2018).

The formation of foam cells occurs in the early phases of AS. The scavenger receptors, such as CD36 and scavenger receptors class A (SR-A) expressed in macrophages combines with oxidized LDL (oxLDL), which is efficiently decomposed and metabolized by lysosomes into free cholesterol and is stored in the cytosol (Collot-Teixeira et al., 2007; Moore and Tabas, 2011; Yu et al., 2013). The accelerated accumulation of cholesterol induces the formation of foam cells. In contrast, cholesterol efflux, by which cholesterol-loaded macrophages within the vessel wall secrete cholesterol outside cells, is understood to be a major process in repressing macrophage conversion (Ohashi et al., 2005). The main transporters of cholesterol efflux include the adenosine triphosphate binding cassette transporters ABCA1/ABCG1 and scavenger receptor class B type I (SR-BI) (Yu et al., 2013). In the New Zealand rabbit AS model, baicalin significantly enhances the expression of ABCA1 and ABCG1 (He et al., 2016). PPARγ is part of a metabolic cascade highly expressed in macrophage that enhances cholesterol efflux, and liver receptor X (LXRα) directly increases the expression of ABCA1 (Chawla et al., 2001). One study found that baicalin inhibited the accumulation of cholesterol and delayed the transformation of macrophages into foam cells, which was the result of the upregulation of the PPAR γ-LXR α-ABCA1/ABCG1 pathway. However, the effects of baicalin on the expression of SR-A and CD36 were not obvious (He et al., 2016). As the high-density lipoprotein (HDL) receptor, SR-BI has been identified as a critical role in promoting cholesterol efflux in macrophages (Gu et al., 2000; Trigatti et al., 2004). In a THP-1 macrophage study, researchers concluded that baicalin induced cholesterol efflux *via* the PPARγ-LXRα-SR-BI pathway (Yu et al., 2016). Furthermore, HDL is reported to be responsible for the reverse transport of cholesterol from the peripheral tissues to the liver for reuse or for the final elimination *via* excretion into the bile. Baicalin is capable of up-regulating the plasma levels of HDL, which implies that increasing HDL might be a mechanism of action for baicalin to expedite cholesterol ejection from macrophages and weaken their conversion (Hovingh et al., 2003; Xi et al., 2015). Taken together, it is rational to propose that baicalin might attenuate AS partly by modulating lipid metabolism and foam cells formation.

#### Inhibition of Oxidative Stress

The imbalance between the overproduction of oxygen reactive species (ROS) in cells and tissues and the ability of a biological system to remove ROS products is called oxidative stress (Peluso et al., 2012; Pizzino et al., 2017). Under pathological conditions, oxidative stress exacerbates the AS. Plasma LDL maybe trapped and accumulate in injured vascular endothelium and be oxidized by ROS to ox-LDL. In addition, the recruited monocytes and migrated lymphocytes can also release large amounts of ROS and which favor the synergism of early atherosclerotic lesions. Moreover, ROS can promote vascular smooth muscle cells (VSMCs) proliferation and collagen deposition thereby leading to the development of an atheromatous plaque in the final (Tousoulis et al., 2015; Kattoor et al., 2017). Recently, considerable data has revealed that baicalin could on one hand attenuate AS by decreasing oxidative stress products and on the other hand strengthen the anti-oxidative system.

Firstly, baicalin may directly or indirectly reduce oxidative stress products. Malondialdehyde (MDA) is a decomposition product of lipid hydroperoxides and can be used as an indicator of oxidative damage to cells and tissues (Bonnes-Taourel et al., 1992). Baicalin was shown to effectively relieved the oxidative stress by abrogating the upregulation of MDA in ApoE-/- mice (Wu et al., 2018). Secondly, in terms of the effects of baicalin on the antioxidative system, the antioxidant enzymes in the vascular wall are not only important radical superoxide scavengers that protect cells from oxidative damage, but are also important parts of the endogenous antioxidant defense system (Olsvik et al., 2005; Ku et al., 2009; Hassan et al., 2014; Wu et al., 2018). The experiment designed by Wu suggested that baicalin treatment significantly relieved the oxidative stress by up-regulating the activities of superoxide dismutase (SOD), catalase (CAT) and glutathione peroxidase (GSH-Px) in a dose-dependent manner compared to the AS model group (Wu et al., 2018). This suggests that baicalin can increase antioxidant enzyme activity and thus improve AS. Further, Nuclear factor E2-associated factor 2 (Nrf2), a basic leucine zipper transcription factor, regulates antioxidant proteins to prevent oxidative damage. As the “master regulator” of the antioxidant response, Nrf2 can modulate the expression of antioxidant enzymes such as heme oxygenase-1 (HO-1) (Loboda et al., 2016). It has been reported that Nrf2/HO-1 pathway exhibits protective roles in many ischemic disorders. Hypoxia stimulates the Nrf2/HO-1 pathway activation in cardiomyocytes (Zeng et al., 2015). Baicalin treatment may enhance the Nrf2 and HO-1 expression and further activate the Nrf2/HO-1 pathway (Yu et al., 2019). Taken together, the potent abilities in elevating antioxidant enzymes by baicalin might lead to the elimination of ROS and subsequently ameliorate oxidative stress-induced atheromatous plaque formation.

#### Mitigation of the Inflammatory Response

AS related inflammation mediated by chemokines, adhesion molecules, proinflammatory cytokines and inflammatory signaling pathways plays an important role in all stages of the atherosclerotic progression (Ross, 1999; Libby et al., 2011; Zhu et al., 2018). Injured endothelial cells activated by inflammatory factors stimulate the biosynthesis of monocyte chemoattractant protein 1 (MCP-1), intercellular adhesion molecule 1 (ICAM-1), vascular adhesion molecule 1 (VCAM-1), and pro-inflammatory cytokines that recruits circulating monocytes to the intima. Migrated monocytes differentiate into macrophages that contribute to inflammation and plaque development (Chistiakov et al., 2018). In an *in vitro* study, baicalin was found to decrease monocyte adhesion *via* reducing the expression of MCP-1, ICAM-1, and VCAM-1 in the presence of high glucose-induced human umbilical vein endothelial cells (HUVECs). Similarly, baicalin had been confirmed to markedly decrease the release of MCP-1, VCAM-1 and IL-6 in the kidney of apolipoprotein E (ApoE)’ knockout (KO) mice (Liu et al., 2015). Further, baicalin inhibited the synthesis of neutrophil chemokines and blocked the biological activity of receptors by selectively binding chemokine receptors, thereby inhibiting the migration and aggregation of inflammatory cells and reducing the degree of AS (Li et al., 2000). Furthermore, in the development of AS-induced inflammation, inflammatory signaling pathways like nuclear factor kappa B (NF-κB) and Wnt1/DKK1 also have a proatherogenetic impact from fatty streak formation to luminal occlusion. It has been reported that the activation of the NF-κB increases the production of inflammatory cytokines and chemokines that promote the progression of AS (Kutuk and Basaga, 2003). Thus, the inhibition of NF-κB signaling has been shown to reduce the incidence of AS. Baicalin also suppressed the activation of NF-κB in human umbilical vein endothelial cells in previous studies (Kim et al., 2006; Ku and Bae, 2015). Another important mechanism of baicalin is mediated by the Wnt1/DKK1 signaling pathway. The Wnt1 signaling pathway is a highly conserved cellular communication system (Peifer and Polakis, 2000). Recent evidence suggests that Wnt signaling participates in inflammatory regulation and the pathogenesis of AS (Sen and Ghosh, 2008). Specifically, Wnt1 plays an anti-atherosclerotic role (Gherghe et al., 2011). Dickkopf-related protein-1 (DKK1), which interacts with Wnt, has recently been considered as a biomarker for AS (Niehrs, 2006; Ueland et al., 2009; Kim et al., 2011). Therefore, the intervention of the Wnt1/DKK1 pathway may represent a feedback approach to resist the progression of AS. A basic study designed by Wang et al. confirmed that baicalin exerted a regulatory effect on inflammation and prevented AS by promoting Wnt1 signally and inhibiting the expression of DKK1 through an elevated Wnt1/DKK1 ratio (Wang et al., 2016).

#### Immunomodulatory Activity

AS is a chronic, low-grade inflammatory response that attracts cells of the innate and adaptive immune systems to the atherosclerotic plaque (Libby, 2002). Experimental and clinical evidence has revealed that both innate and adaptive immunity play important roles in the onset and progression of AS (Wolf and Ley, 2019). The complex network of interactions among vascular components and immune cells regulate the balance between proatherogenic inflammatory and atheroprotective anti-inflammatory responses (Herrero-Fernandez et al., 2019).

The natural immune cells involved in AS mainly include mononuclear-macrophages and dendritic cells (DCs) (Shimada, 2009; Hansson and Hermansson, 2011; Ammirati et al., 2015). Circulating monocytes and resident macrophages are the earliest recruited to atherosclerotic plaques and the most abundant immune cells. Baicalin may prevent the proliferation of mononuclear cells and inhibit macrophage activation (Liu et al., 2008). Macrophages can be divided into classically activated pro-inflammatory (M1) and alternatively activated anti-inflammatory (M2) phenotypes (Tan et al., 2016). M2_C_ is one subtype of M2 macrophages and is mainly responsible for the phagocytosis of apoptotic cells (Zizzo et al., 2012). RAW264.7 macrophages in the M1 phenotype induced by lipopolysaccharide (LPS) could easily be switched to M2 phenotype after baicalin induction. Thus, baicalin may promote M2_C_ macrophages polarization and increase the atherosclerotic plagues clearance (Lai et al., 2018). Previous study showed that baicalin could significantly increase the serum levels of interleukin (IL)-10 and transforming growth factor-β1 (TGF-β1) (Liao et al., 2014). Meanwhile, the up-regulated expression of IL-10 might be involved in reducing tissue migration of neutrophils, boosting the formation of regulatory T cells and promoting phagocytic activity of M2c (Van den Akker et al., 2013; Lai et al., 2018). Other studies had also shown that baicalin promoted M2 macrophage polarization *in vitro* by increasing interferon regulatory factor 4 (IRF4) protein expression and by decreasing M1 markers such as tumor necrosis factor α (TNF-α), IL23, and IRF5 (Zhu W. et al., 2016). In an LPS-induced rat model, baicalin could also repress the toll-like receptor (TLR)4 signaling pathway of the peripheral blood mononuclear cells (Ye et al., 2015). Furthermore, DCs, presenting oxLDL-derived antigens in atherosclerotic plaques and secondary lymphoid organs, could treat oxLDL in plaques and stimulate adaptive immune responses (Steinman et al., 1975; Steinman and Hemmi, 2006). Liu et al.’s research pointed out that baicalin inhibited the expression of the CD11c marker on DCs in aorta tissue and CD11C, CD83, CD80, and CD86 markers on DCs in the bone marrow. Thus, baicalin may have exert immune-regulatory effects and prevent the formation of atherosclerotic lesions by decreasing the DC numbers, and inhibiting DC maturation in bone marrow and infiltration into lesions (Liu et al., 2014). Baicalin can also affect DC-related inflammatory mediators, especially the expression of IL-12.

The adaptive immune system is also involved in AS progression. CD4^+^ T cells are the key regulatory cells for adaptive immune response and have the ability to differentiate into different T helper subtypes including atherosclerotic T cells (TH1 and TH17 cells) and protective cells (TH2 and regulatory T [Treg] cells) Among these, TH17 secretes signature cytokine IL-17A and has a role in triggering immune inflammation and promoting AS. Natural Treg cells (nTreg cells) express factor forkhead box protein P3 (Foxp3) and can negatively control immune responses (Foks et al., 2011; Tse et al., 2013; Song and Yang, 2017; Gisterå and Hansson, 2017). Ample literature has documented there is a Th17/Treg imbalance in patients with coronary artery AS, accompaned by a significant increase in Th17 and a decrease in Treg cells (Li et al., 2014; Potekhina et al., 2015). There is an increasing effort to identify immune-modulating therapies targeting immune cells with a potential anti-atherosclerotic impact. Convincing evidence indicates that baicalin could exert its anti-arteriosclerosis effects mainly by balancing Th17/Treg cells. As mentioned before, Foxp3 could release anti-inflammatory cytokines such as IL-10 and TGF-β1, which plays a crucial role in the differentiation of Treg cells (Marson et al., 2007; Ait-Oufella et al., 2009). There is an investigation indicating that baicalin could markedly induce Foxp3 expression and increase Treg cells, as well as increase the levels of two relational serum cytokines (TGF-β1 and IL-10) in animal models (Liao et al., 2014). Similarly, Yang et al. found that baicalin up-regulated both exogenous and endogenous Foxp3 expression and promoted Treg cell differentiation *in vitro* using HEK 293T cells as a cell model (Yang et al., 2012). In their previous studies, the same authors also confirmed that baicalin could inhibit the differentiation of Th17 cells *in vivo* and *in vitro*. The mechanism might be closely associated with baicalin inhibition the IL-6 and RORctγ expression (Yang et al., 2011). Baicalin works by regulating the immune balance of Th17/Treg cells. The above evidences support the concept that baicalin can improve AS through immunomodulation, which may represent an important mechanism underlying the anti-atherosclerosis effects of baicalin. Apart from influencing adaptive immune cells, baicalin had been reported to also inhibit the production of red blood cell immunity, especially IgG production, by regulating the Treg/Th17 axis, but the correlation between red blood cell immunity with AS is unclear (Jiang et al., 2019). In short, the therapeutic utility of baicalin in AS at least partly ascribed to the regulation of immunomodulatory effects.

#### Suppression of VSMC Proliferation and Migration

Abnormal proliferation and migration of vascular smooth muscle cells (VSMCs) lead to intimal focal fibrous thickening and atherosclerotic plaque formation, while it is worth noting that VSMCs in advanced plaques are entirely beneficial. For example, VSMCs preventing rupture of the fibrous cap. Smooth muscle (SM)22, a differentiated VSMC marker, is a cytoskeleton-associated protein and is important for maintaining the differentiated phenotype of VSMCs (Fu et al., 2000; Han et al., 2009; Dong et al., 2012). Disruption of SM22α is known to increase atherosclerotic lesions and enhance arterial pro-inflammation (Shen et al., 2010; Shu et al., 2015). Baicalin has been reported to up-regulate the SM22α which may represent a safe and effective approach to prevent vascular disease (Lv et al., 2016). Moreover, baicalin is able to inhibit the proliferation of VSMCs by repressing cell cycle progression and arresting the human aorta VSMCs cycle at the G0/G1 phase. The molecular mechanism of this effect was associated with activating the expression of maternally expressed gene 3 (MEG3), which is a long-chain non-coding RNA, whose transcriptional deficiency increases cell cycle-associated protein expression (Liu et al., 2019). Furthermore, baicalin could activate the p53 signaling pathway and promoted the expression and transport of p53 leading to apoptosis of VSMCs. Overall, based on an *in vitro* model of AS, baicalin inhibited proliferation and promoted apoptosis in ox-LDL-treated HA-VSMCs by activating the expression of MEG3/p53 (Liu et al., 2019). Furthermore, baicalin has also been reported to suppress the proliferation and migration of ox-LDL-VSMCs by upregulating a family of endogenous, small and non-coding RNAs called miR-126-5p by targeting high-mobility group box 1 (HMGB1) (Chen et al., 2019). Thus, it is apparent that blockade of VSMC proliferation and migration are important constituents of the atheroprotective effects of baicalin.

### Myocardial Ischemia–Reperfusion Injury (MIRI)

Ischemic heart disease is primarily caused by coronary AS and its complications. MI is the most common cause of ischemic heart disease (Pasupathy et al., 2015). Reperfusion is mandatory to salvage the ischemic myocardium from infarction, but reperfusion *per se* contributes to injury and ultimate infarct size (Reimer et al., 1993; Vandervelde et al., 2006; Fernández-Jiménez et al., 2015). Therefore, cardioprotection beyond that by timely reperfusion is needed to reduce infarct size and to improve the prognosis of patients with acute myocardial infarction. Numerous studies have confirmed that baicalin has a protective effect on the infarcted myocardium involved in myocardial infarction and myocardial ischemia-reperfusion, and its main mechanism of action includes inflammation regulation, inhibition of oxidative stress and reduction of apoptosis (Lin et al., 2010; Kong et al., 2014).

#### Mitigation of Inflammatory Response

MI initiates an intense inflammatory response, while inflammation is an important process in the pathophysiological myocardial I/R injury (Frangogiannis, 2014; Ma et al., 2014; Yang et al., 2016). Under inflammatory conditions, signaling pathways like mitogen-activated protein kinases (MAPKs) and NF-κB are activated, aggravating large amounts of pro-inflammatory markers such as TNF-α, IL-1β, IL-6, and IL-18, and anti-inflammatory cytokines such as IL-10 which protect cardiac function (Chandrasekar et al., 2000; Hall et al., 2006). MAPK cascades, especially extracellular signal-regulated kinase (ERK), c-Jun N-terminal kinase/stress-activated protein kinase (JNK/SAPK) and p38, are expresses in the myocardium and play a pivotal role in the amelioration of ischemic insults (Yue et al., 2000; Petrich and Wang, 2004). Current work has shown that the phosphorylated (p)-ERK was reduced while the p-JNK and p-p38 were elevated in the rat model of acute MI (Liu et al., 2013). Therefore, targeting the inflammatory cascade and related inflammatory cytokines is crucial in improving myocardial ischemia and reperfusion.

Baicalin plays a protective role in ischemia/reperfusion (I/R) myocardial injury and protects against hypoxia/reoxygenation (H/R) damage. A previous report confirmed that baicalin protected against I/R injury in cultured chick cardiomyocytes (Chang et al., 2006). In terms of the baicalin on the anti-inflammatory mechanisms, on one hand, baicalin’s cardioprotection was associated with mediation of MAPKs cascades. In the acute MI (AMI) model of rats, which following treatment with baicalin treatment, Liu and colleagues found that the cardioprotective effect of baicalin might be achieved *via* the activation of ERK and suppression of JNK and p38 activity. They also showed that baicalin played a favorable role against AMI impairment by decreasing myocardial injury marker such as the creatine kinase (CK), creatine kinase MB (CK-MB), lactate dehydrogenase (LDH) and serum cardial troponinT (cTnT) as well as reducing the infarction size (Liu et al., 2013). Similarly, results from the MI rats model induced by isoproterenol, not only did baicalin ameliorated infarct size and CK, CK-MB, LDH and cTnT levels, but it also suppressed p-38 protein expressions significantly (Sun et al., 2015). Conversely, baicalin treatment effectively inhibited the NF-κB pathway and exerted cardioprotective effects in the cultured rat cardiomyocytes exposed to H/R (Lin et al., 2010). The phosphatidylinositol 3-kinase (PI3K)/Akt signaling pathways also participates in the regulation of NF-κB in the inflammatory response. In the male rats I/R model, intragastric administration of baicalin could attenuate I/R-induced myocardial damage *via* activating PI3K/Akt signaling and suppressing NF-κB signaling (Luan et al., 2019). In addition, among these studies, regulation of the levels of inflammatory cytokines was almost consistent. With the treatment of baicalin, pro-inflammatory markers such as TNF-α, IL-6, IL-1β and IL-8 in myocardial tissues were down-regulated, and the anti-inflammatory cytokine, IL-10, was up-regulated. Thus, according to the above findings, it is apparent that baicalin may directly suppress the inflammatory response and may then improve inflammatory-elicited myocardial injury.

#### Inhibition of Oxidative Stress

At least half of myocardial damage resulting from MI is associated with myocardial reperfusion injury. Once the blood supply to an organ is interrupted (ischemia) and re-established (reperfusion), this kind of situation leads to a “burst” of ROS generation from mitochondria including uncoupled SOD and MDA (Cadenas et al., 2010; Hausenloy and Yellon, 2013). There is evidence indicating that baicalin can improve ventricular function on I/R injury in isolated rat hearts, the mechanisms of which may be associated with increasing SOD and decreasing MDA activity (Kong et al., 2014). Furthermore, in a rat model of MI, a decrease in MDA and an increase in of SOD were observed in the baicalin treatment group (Wang L. et al., 2018). Therefore, baicalin supplementation should be considered as an effective approach for attenuating reperfusion-exerted microvascular damage. In addition, Nrf2, a redox-sensitive transcription factor, is crucial to inhibit oxidative stress in cells (Gallorini et al., 2015). Once activated, Nrf2 can transactivate genes driven by antioxidant response elements (ARE), especially HO-1 (Tong and Zhou, 2017). Herein, it has been reported that the Nrf2/HO-1 pathway exhibits protective roles in ischemic disorders, including MI (Zeng et al., 2015). One *in vitro* study demonstrated that baicalin treatment activated the Nrf2/HO-1 pathway in H9c2 cells under hypoxic condition *via* further enhancing Nrf2 and HO-1 expression (Yu et al., 2019). Taken together, baicalin may be a potential preventive and therapeutic compound for improving MI.

#### Anti-Apoptosis Activity

Apoptosis, is involved in MI evolution, prognosis and outcome, constitutes an important form of cardiac cell death after MI (Akasaka et al., 2006; Chen et al., 2014). Inhibition of myocardial cell apoptosis can reduce the I/R injury, which is one of the clinical significance for the treatment of MI (Luan et al., 2019). Baicalin pretreatment protects against myocardial I/R injury by inhibiting mitochondrial damage-mediated apoptosis. Baicalin also exerts cardioprotective effects *via* regulating several signaling pathways and apoptosis regulators. The Wnt/β-catenin signaling pathway is associated with ischemic heart disease. Knockdown of β-catenin expression inhibited H2O2-induced cardiomyocyte apoptosis. Baicalin could significantly downregulate the expression of β-catenin in H2O2-treated H9c2 cells. It was speculated that baicalin might inhibit the Wnt/β-catenin signaling pathway and thus inhibit cell apoptosis, allowing it to exert a cardioprotective role. However, whether it is directly related to MI and reperfusion remains to be verified (Zhang et al., 2009; Gessert and Kühl, 2010; Dohn and Waxman, 2012; Qiu et al., 2017). The PI3K/Akt may also be regulated by baicalin to inhibit I/R-induced cardiomyocyte apoptosis thereby reduction of myocardial damage. Caspase-3 is an important apoptosis executors in the caspase family, and up-regulating the expression of caspase-3 gene promotes AMI myocardial apoptosis (Zidar et al., 2007; Prech et al., 2010; Chen et al., 2014). Cell-based research indicated that baicalin could inhibit caspase-3 activity and protein expression to effectively protect the heart from MI damage. The specific mechanism involved might be related to the activation of survival kinases including ERK and the inhibition of apoptotic kinases such as JNK and p38 (Sun et al., 2015). Bax and Bcl-2 belong to the same apoptosis gene family, but they have opposite effects. Bcl-2 inhibits cell apoptosis, while Bax promotes it (Karsan et al., 1997). Baicalin could improve MI by up-regulating the expression of Bcl-2 and down-regulating Bax (Wang L. et al., 2018). Similarly, the results of another *in vitro* experiments also implied that pretreatment with baicalin significantly reduced cytochrome c, Bax and increased Bcl-2 expression in H/R-induced cardiomyocytes (Jiang et al., 2018).

Furthermore, mitochondrial injury-mediated apoptosis is also an important baicalin mechanism potentially able to protect against myocardial I/R injury. It is well known that cardiomyocyte apoptosis, resulting from mitochondrial dysfunction, is considered to be a major contributor to I/R injury (Wang et al., 2013). The most representative is mitochondrial aldehyde dehydrogenase 2 (ALDH2), which is widely expressed in the heart, is an anti-apoptotic enzyme that participates in oxidative stress-induced cell apoptosis (Zhang et al., 2011; Sun et al., 2014). ALDH2 maintains mitochondrial function and inhibits ROS generation (Vander Heide and Steenbergen, 2013). Emerging evidence has revealed that ALDH2 has a cardioprotective role in myocardial IR injury (Pang et al., 2015). The study by Jiang et al. demonstrated that baicalin could reduce the apoptosis and oxidative stress by enhancing the expression and activity of ALDH2 to protect against the cardioprotective effect of H/R-induced H9c2 cell damage (Jiang et al., 2018). Taken together, understanding the effects of baicalin in mediating functions of anti-apoptotic activity would supply newly insights into the understanding for the treatment of MIRI.

### Hypertension

Hypertension is a complex disease that involves an interaction between environmental factors, genetic influences and abnormalities in regulatory mechanisms of the cardiovascular system (Parati, 2015; Hoffmann et al., 2017; Calvillo et al., 2019). Cumulative findings support the notion that inflammation, oxidative stress and endothelial dysfunction leads to the development of hypertension (Dinh et al., 2014). Targeting these pathogenesis processes is indicated to be a critical mechanisms underlying the baicalin hypotensive effect and subsequent cardiovascular events (Ding et al., 2019).

Recently, the immune system and inflammatory response have been shown to play an essential role in the pathogenesis of hypertension. Many inflammatory markers such as C-reactive protein (CRP), cytokines, and adhesion molecules have been found to be elevated in hypertensive patients supporting the role of inflammation in the pathogenesis of hypertension. CRP could stimulate monocytes to release proinflammatory cytokines such as IL-6, IL-1β, and TNF-α which will further promote inflammation (Crowley, 2014). *In vivo*, experimental data confirmed that baicalin forcefully lowered the blood pressure partly *via* decreasing the serum levels of high-sensitivity CRP, IL-6 and IL-1β in spontaneously hypertensive rats (SHRs) (Wu et al., 2019). Similarly, another vitro study showed that baicalin extenuated contents of TNF-α by altering miR-145 expression and significantly ameliorating TNF-α inflammatory injuries in human aortic endothelial cells (HAECs) (Li et al., 2020). With the popularization of the Holistic concept, numerous studies have revealed that a damage in the intestinal barrier, is considered as a critical role in the pathogenesis of hypertension primarily by exacerbating the development of chronic low-grade inflammation (Santisteban et al., 2017; Jaworska et al., 2017). The increment of intestinal permeability caused by intestinal barrier dysfunction allows leakage of bacterial metabolites into the circulation. One such metabolite is bacterial lipopolysaccharide, a TLR4 ligand, which is increased in the circulation of patients with hypertension (Kim et al., 2018). One of an important consequence of TLR signaling is the priming of inflammasomes. Various inflammasome-derived cytokine IL-1β or/and IL-6 is augmented in several blood pressure-regulating sites during hypertension, including medial and adventitial layers of arteries (Krishnan et al., 2016; Zhu Q. et al., 2016). Existing study proves that baicalin take the protective effects on the intestinal integrity in the SHRs. One molecular evidence is that baicalin could decreases the serum levels of inflammatory indicators such as high-sensitivity C-reactive protein (hs-CRP), IL-1β, and IL-6. Another one is that baicalin increases the expression of tight junction proteins such as zonules occludin-1 (ZO-1), cingulin and occluding. Besides, short-chain fatty acids (SCFAs), as the metabolites of intestinal microbes, also play an important role in the integrity of the intestines, and their pathway of action may be related to enhancing the expression tight junction proteins (Wu et al., 2019). A pivotal study shows that baicalin treatment blunts the development of experimental hypertension partly by promoting the production of fecal SCFAs and the abundance of SCFA-producing bacterial in the SHRs. Thus, all these studies support the protective effects of baicalin on the intestinal integrity in the treatment of hypertension. According to previous studies, sustained inflammation contributes to the overproduction of ROS which in turn aggravates the inflammatory process and perturbs the function of the vasculature (Crowley, 2014). In the pathogenesis of hypertension, Angiotensin II (Ang II), as the main active peptide of renin-angiotensin system (RAS), can participate in oxidative stress and apoptosis leading to endothelial dysfunction by activating apoptosis-related proteins, stimulating ROS production, and reducing NO production. Therefore, interfering with Ang II-induced endothelial dysfunction, inhibiting oxidative stress, and reducing cell apoptosis can effectively improve hypertension. A study involving the HUVEC model of Ang II injury determined that baicalin can significantly reduce the endothelial dysfunction and oxidative stress induced by Ang II. These positive effects are mainly achieved by regulating the expression of Bax, Bcl-2 and cleaved caspase-3, activating the ACE2/Ang-(1-7)/mas axis, and upregulating the PI3K/AKT/eNOS pathway. They also found that baicalin attenuated oxidative stress indicators such as reducing MDA and ROS, promoting nitric oxide (NO) and Total Antioxidant Capacity (T-AOC) levels (Heitsch et al., 2001; Mehta and Griendling, 2007; Sampaio et al., 2007; Wei et al., 2015). Another potential mechanism involved in the hypertension-protecting effects of baicalin is enhancing endothelial nitric oxide synthase (eNOS) -induced production of endogenous NO in HUVECs (Chen et al., 2013). Apart from the above pathogenetic mechanisms involving baicalin, baicalin lowered blood pressure partially by relaxing vascular smooth muscle by decreasing Ca^2+^ levels and the enhancing K_ATP_ function in VSMCs (Ding et al., 2019). The combined application of baicalin and berberine was also found to relax blood vessels, owing to the voltage-dependent Ca^2+^ channel (VDCC) (Wu et al., 2020). Together, these studies indicated that baicalin might have comprehensive effects on ameliorating hypertension.

### Heart Failure

**Figure 4:**
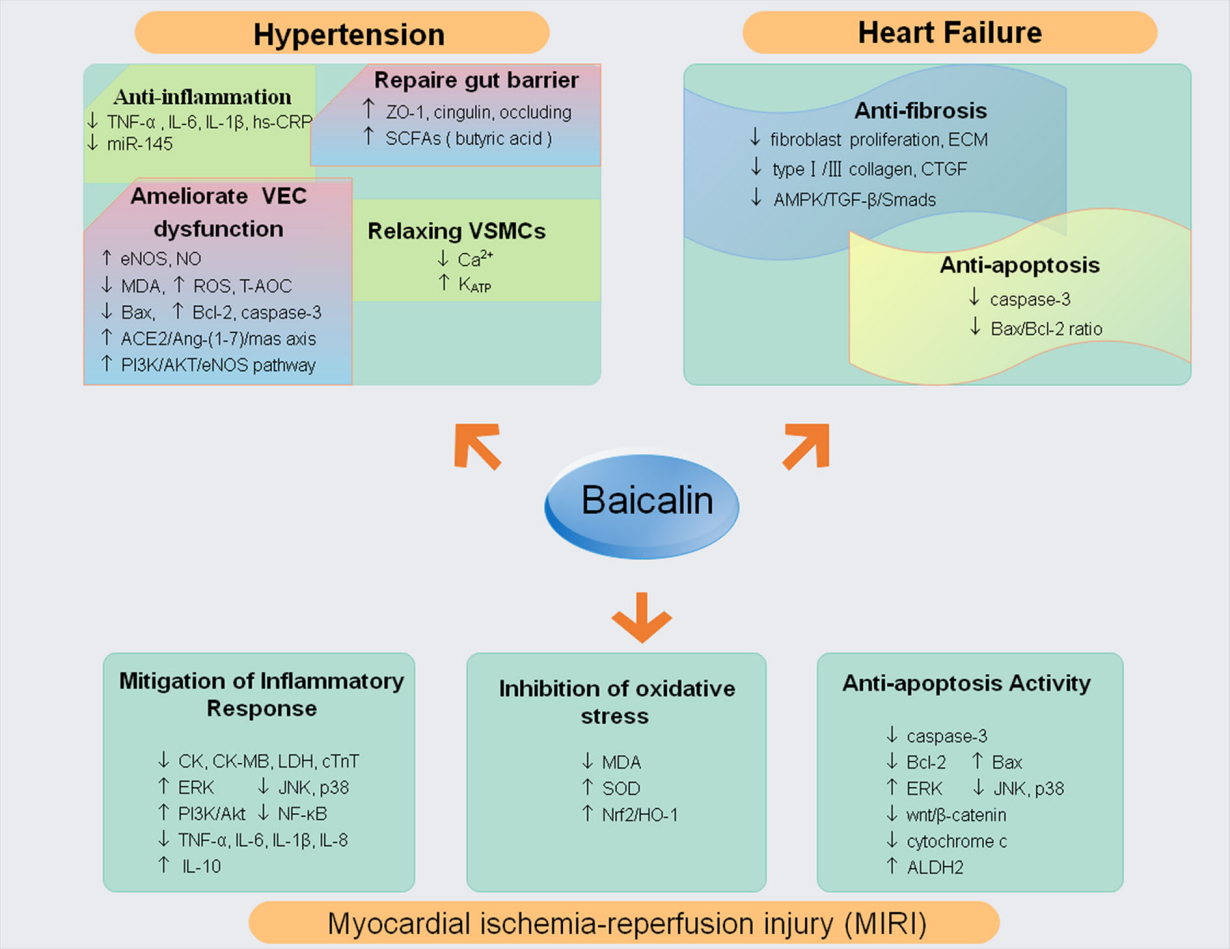
The molecular mechanisms and potential targets of baicalin in hypertension, myocardial infarction and reperfusion, heart failure (↓ decrease or inhibit; ↑ increase or up-regulate). Abbreviations: tumor necrosis factor (TNF)-α; extracellular signal-regulated kinase (ERK); c-Jun N-terminal kinase (JNK); phosphatidylinositol 3-kinase (PI3K); nuclear factor kappa B (NF-κB); interleukin 6, 8, 10, 1β (IL-6, IL-1β, IL-8,IL-10); superoxide dismutase (SOD); malondialdehyde (MDA); nuclear factor E2-associated factor 2 (Nrf2); heme oxygenase-1 (HO-1); mitogen-activated protein kinases (MAPKs); mitochondrial aldehyde dehydrogenase 2 (ALDH2); high-sensitivity C-reactive protein (hs-CRP); zonula occludens-1 (ZO-1); Short-chain fatty acids (SCFAs); oxygen reactive species (ROS);(eNOS);Total Antioxidant Capacity (T-AOC); nitric oxide (NO); extracellular matrix (ECM); Connective Tissue Growth Factor (CTGF); creatine kinase (CK), creatine kinase MB (CK-MB), lactate dehydrogenase (LDH) and serum cardial troponinT (cTnT); malondialdehyde (MDA); superoxide dismutase (SOD).

Heart failure (HF) is the final process resulting from different cardiac insults and subsequent dysregulation of in compensatory mechanisms and pathogenic processes (Yancy et al., 2013). Cardiac fibrosis characterized by interstitial fibroblast proliferation and excessive production and deposition of myocardial extracellular matrix (ECM) proteins is an independent and predictive risk factor for HF in both ischemic and nonischemic cardiomyopathy (Fan et al., 2012; Prabhu and Frangogiannis, 2016). Some studies have revealed that baicalin had an anti-fibrosis effect. One study had observed that baicalin could alleviate myocardial fibrosis manifested by reducing the ECM and decreasing fibrosis genes expression [type I collagen, type III collagen and Connective Tissue Growth Factor (CTGF)] in pressure overload mouse model (Zhang et al., 2017). Similarly, another study found that baicalin could inhibit fibroblast proliferation and ECM accumulation, thereby suppressing cardiac fibrosis from the pressure overload-induced in the abdominal aortic constriction (AAC) rat model; the underlying mechanisms are linked to the AMPK/TGF-β/Smads signaling pathway (Xiao et al., 2018). Moreover, baicalin inhibited apoptosis by reducing the Bax/Bcl-2 ratio and caspase-3, indicating that that suppression of apoptosis could decrease adverse remodeling and subsequent progression to HF (Wencker et al., 2003; Dai et al., 2017). It is worth exploring additional more mechanisms of baicalin-mediated HF protection furtherly (**Figure 4**).

## Conclusion

In conclusion, numerous preclinical studies have provided evidence that baicalin, a naturally occurring bioactive compound in *S. baicalensis Georgi*, is a promising therapeutic agent for cardiovascular protection. The pharmacokinetics profile of baicalin mainly includes gastrointestinal hydrolysis, enterohepatic recycling, carrier-mediated transport and complicated metabolism. A comprehensive understanding of its pharmacokinetics is essential for its safety and efficacy in clinical applications. Baicalin exerts prophylactic and/or therapeutic effects in cardiovascular disorders *via* mechanisms involving in regulating lipid metabolism, reducing inflammation-induced damage, inhibiting oxidative stress, reducing apoptosis, and immune regulation. The pleiotropic pharmacological activities of baicalin suggest it has great potential for clinical application in the prevention and treatment of AS, MIRI, hypertension, and HF. Although a great deal of knowledge has been acquired regarding the benefits of baicalin on CVDs from experimental data, it is worth noting that the specific underlying mechanisms are still relatively unexplained. Thus, the limited clinical and pharmacological data available are not enough to evaluate its efficacy at the present moment. Further, concerning the undesirable physical characteristics of baicalin, additional research and technological development are required to improve bioavailability and to overcome the challenges in its clinical application. Therefore, making full use of modern analytical techniques, establishing reasonable detection methods, and studying the role of baicalin in a more systematic and in-depth manner are important. Further exploration into the molecular mechanisms and potential targets of baicalin, is also urgent and necessary in order to conduct a large randomized and controlled trials to evaluate the efficacy and safety of the cardiovascular activity of baicalin.

## Author Contributions

LY-X wrote the main text. JL-G contributed equally to this work. HC-L, YQ, CS, and YL-W retrieved and organized documents. YD-L and XN-C had great contribution in second time revision, polishing manuscript, and helping in revising figures.

## Funding

This work was funded by grants from the National Natural Science Foundation of China (No.81973842).

## Conflict of Interest

The authors declare that the research was conducted in the absence of any commercial or financial relationships that could be construed as a potential conflict of interest.
